# Corneal pulsation and biomechanics during induced ocular pulse. An ex-vivo pilot study

**DOI:** 10.1371/journal.pone.0228920

**Published:** 2020-02-13

**Authors:** Maja M. Rogala, Daniel Lewandowski, Jerzy Detyna, Agnieszka Antończyk, Monika E. Danielewska

**Affiliations:** 1 Department of Mechanics, Materials Science and Engineering, Wrocław University of Science and Technology, Wrocław, Poland; 2 Department of Surgery, Wrocław University of Environmental and Life Sciences, Wrocław, Poland; 3 Department of Biomedical Engineering, Wrocław University of Science and Technology, Wrocław, Poland; Keio University School of Medicine, JAPAN

## Abstract

The purpose of this study was to ascertain the relationships between the amplitude of the corneal pulse (CP) signal and the parameters of corneal biomechanics during *ex-vivo* intraocular pressure (IOP) elevation experiments on porcine eyes with artificially induced ocular pulse cycles. Two experiments were carried out using porcine eyes. In the first one, a selected eye globe was subjected to three IOP levels (15, 30 and 45 mmHg), where changes in physical ocular pulse amplitude were controlled by infusion/withdrawal volumes (ΔV). In the second experiment, six eyes were subjected to IOP from 15 mmHg to 45 mmHg in steps of 5 mmHg with a constant ΔV, where corneal deformation parameters were measured using Corvis ST. In both experiments, at each IOP, the CP and IOP signals were acquired synchronically using a non-contact ultrasonic distance sensor and a pressure transmitter, respectively. Based on the amplitudes of the CP and IOP signals *ocular pulse based corneal rigidity index* (OPCRI) was calculated. Results indicate positive correlations between ΔV and the physical ocular pulse amplitude, and between ΔV and the corneal pulse amplitude (both *p* < 0.001). OPCRI was found to increase with elevated IOP. Furthermore, IOP statistically significantly differentiated changes in OPCRI, the amplitudes of CP and IOP signals and in most of the corneal deformation parameters (*p* < 0.05). The partial correlation analysis, with IOP as a control variable, revealed a significant correlation between the length of the flattened cornea during the first applanation (A1L) and the corneal pulse amplitude (*p* = 0.002), and between A1L and OPCRI (*p* = 0.003). In conclusion, this study proved that natural corneal pulsations, detected with a non-contact ultrasonic technique, reflect pressure-volume dynamics and can potentially be utilized to assess stiffness of the cornea. The proposed new rigidity index could be a simple approach to estimating corneal rigidity.

## Introduction

In recent years, corneal biomechanics has become of particular interest for improving refractive surgeries [1,2], better understanding pathogenesis of corneal diseases such as keratoconus [3–5] and eye diseases linked to intraocular pressure (IOP) elevation experienced in glaucoma [6–8]. Cornea, like many other soft tissues, represents viscoelastic model of the material [9,10]. The dynamic deformation response of the cornea is described by its viscoelastic properties and originates mainly from molecular rearrangement as a response to the mechanical load application [11,12]. Non-linear stress-strain relationship, described in the literature, indicates that the mechanical properties of the cornea change with the applied stress [13,14]. The cornea, as an integral part of the outer ocular coat, is directly subjected to the internal load of the IOP [13,15]. Thus, as it was reported earlier [7,16–18], IOP itself affects stiffness of the ocular shell. In turn, the corneal mechanical resistance influences IOP fluctuations, e.g. Ocular Pulse Amplitude (OPA) and IOP spikes, as it has recently been proven in both *in-vivo* [7,19] and *ex-vivo* studies [20].

Current efforts are notably focused on developing an accurate and at the same time non-invasive *in-vivo* methodology for determining biomechanical properties of the cornea, which have a considerable clinical relevance. Here, one can specify air-puff systems for the dynamic corneal response evaluation with ultra-high-speed Scheimpflug camera [21–24] or OCT [25–28], Brillouin microscopy [29,30] and ocular pulse elastography [31]. The first is the only commercially available solution, which makes it the one most commonly used, however, the last one is particularly interesting because it aims at involving natural corneal deformation due to the ocular pulse (OP) phenomenon [32]. In general, OP characterizes pulse-synchronous eye volume changes resulting from the pulsatile variations in IOP [33], closely linked to the blood circulation [7,34] and aqueous humor dynamics [35].

Numerous efforts have been made in human *in-vivo* studies to investigate the influence of corneal biomechanical properties on IOP measurements [21,36,37], however, without any knowledge about the true IOP. Studies that considered comparing IOP measurements with the manometrically determined IOP are limited, mainly because of a limited number of patients and ethical restrictions [38,39]. On the other hand, the effect of corneal biomechanics on the accurate IOP readings can be estimated using an appropriate mathematical model, such as that proposed by Liu and Roberts [40]. *Ex-vivo* studies, however, provide an opportunity to link estimated IOP values to true IOP by taking into account controlled corneal biomechanical properties [16,41,42]. Including OP cycles simulations to such experiments can be a valuable source of additional knowledge about the corneal biomechanics intrinsic to the ocular dynamics. To the best of our knowledge, the first *ex-vivo* test with OP cycles simulation was performed very recently by Liu [32]. The applied methodology combined the advantage of *in-vivo* tests (a possibility to closely reflect the real pulsatile IOP conditions) with the advantage of *ex-vivo* tests (the ability to fully control those conditions). Those *ex-vivo* OP simulations gave the opportunity to thoroughly investigate the IOP dynamics simultaneously with the biomechanical changes of cornea.

Similarly, parameters of ocular dynamic due to OP have been assessed via examining the natural corneal pulse (CP) defined as a superposition of slight semi-periodic corneal surface expansion and longitudinal eye globe movements [43–45]. Measurements of the CP signal, registered with non-contact and non-invasive ultrasonic technique [46], were performed both in humans [45,47–50], and in animals [51]. The advantage of this technique is the ability to measure the natural pulsation of the cornea without using any external mechanical excitation system that would affect natural tissue dynamics. In our earlier study, in anesthetized rabbits [51], the CP signal’s parameters were referred to true IOP, however, without controlled corneal biomechanical properties at each IOP value. Combining elements of either the CP cycles and corneal biomechanics for different IOP conditions can bring new insight into their mutual dependencies.

The aim of this study was to ascertain the relationships between the corneal pulse amplitude and the parameters of corneal biomechanics during the *ex-vivo* IOP elevation experiments on porcine eyes with artificially induced OP cycles.

## Materials and methods

### Specimens preparation

Porcine eye globes were obtained from the local abattoir Dworecki’s Meat Processing Plant (Golejowo, Poland) at most 6 h post-mortem and tested within the maximum of 12 h post-mortem, similarly as in the work of Kling et al [52]. The residues of the eye muscles were mechanically separated from each eye, then the eye globes were placed in a storage medium of phosphate-buffered saline (PBS) solution at 4°C before the experiment began. Before qualifying porcine eye globes for the measurement, the corneas were carefully checked using a slit lamp in order to assess existing edema, endothelial damage, mechanical damage and corneal transparency. The inclusion criteria were: lack of mechanical damages of the cornea, conjunctiva and sclera, corneal transparency remaining throughout the entire procedure, optic nerve intact, and central corneal thickness (CCT) ranging between 950 μm and 1200 μm. This narrow range was used to ensure that eye globes have corneas of similar thickness, because it can influence the biomechanical properties of the tissue [53], and hence the ability of cornea to deform during the ocular pulse simulation.

Each eye globe underwent the same procedure. First, an eye was carefully placed in a custom-made holder internally padded with PBS-moistened cotton. Restraint of the whole eye movement was obtained by gently binding the optic nerve to the holder with the polyamide sewing thread. The thread was slightly tensed during the measurements so that the eye globe was lightly pressed to the rear wall of the holder. Then, optical biometry was performed using the IOL Master 700 (Carl Zeiss Meditec AG, Jena, Germany) to ensure uniformity of the samples in terms of their geometrical parameters and to exclude outliers. In order to maintain proper hydration of the cornea during the measurement, a few drops of Eusol-C (Alchimia, Ponte San Nicolo, Padova, Italy) were applied to corneal surface.

### Dynamic inflation experiment with induced OP

**Fig 1:**
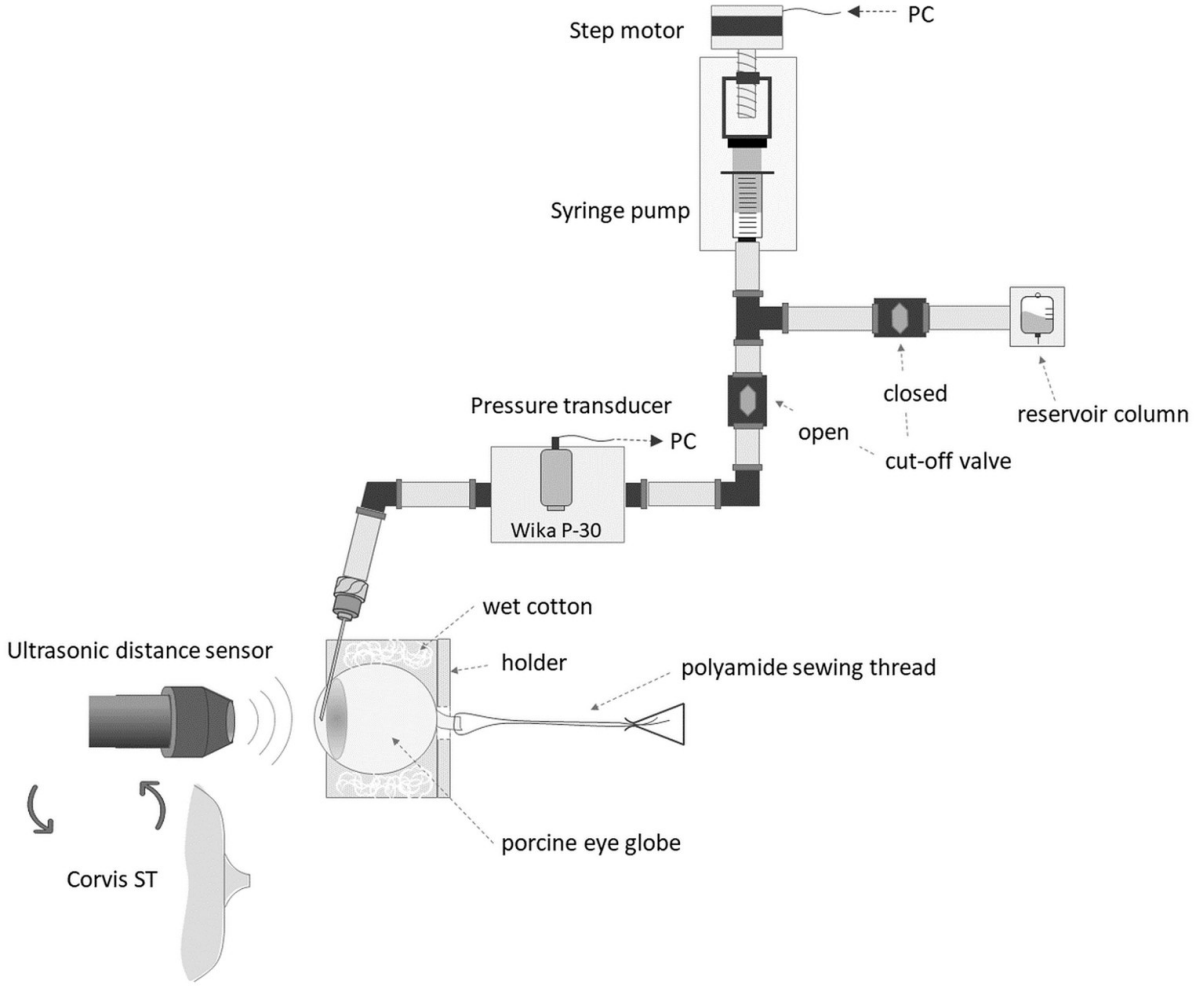
A scheme of the inflation test rig for the CP signal measurement during induced ocular pulse (OP) cycles and for corneal biomechanical measurements.

Dynamic behavior of the corneal tissue was investigated during inflation test. From that moment, a mineral oil was applied to the eye globe surface to prevent loss of hydration. This procedure was also aimed at prevention of the tissue swelling [54]. The 19G injection needle was inserted into the anterior chamber of the eye from the corneoscleral limbus area in order to control the value of the intraocular pressure (IOP) and to provide the pressure fluctuations simulating the OP. This relatively large size of the needle was selected to avoid pressure loss and prevent delays in the experimental setup. The needle was attached to the pressure transmitter P-30 (WIKA, Germany) of relative pressure range 0–187.5 mmHg (0.05% accuracy class) and to the custom-made syringe pump via non-deformable polytetrafluoroethylene tubing and the set of quick couplings, all filled with PBS and free of air bubbles (Fig 1). The software consisted of two parts. The first one directly controlled the work of the pump motor. It operated on a microprocessor (Teensy 3.5, MK64FX512VMD12, 120 MHz, ARM Cortex-M4a and FPU) with codes written in C language. The second part, supervising the pumping process and communicating microprocessor with the PC, was written using Keysight VEE (Pro 9.3, Keysight Technologies Inc.).

**Fig 2:**
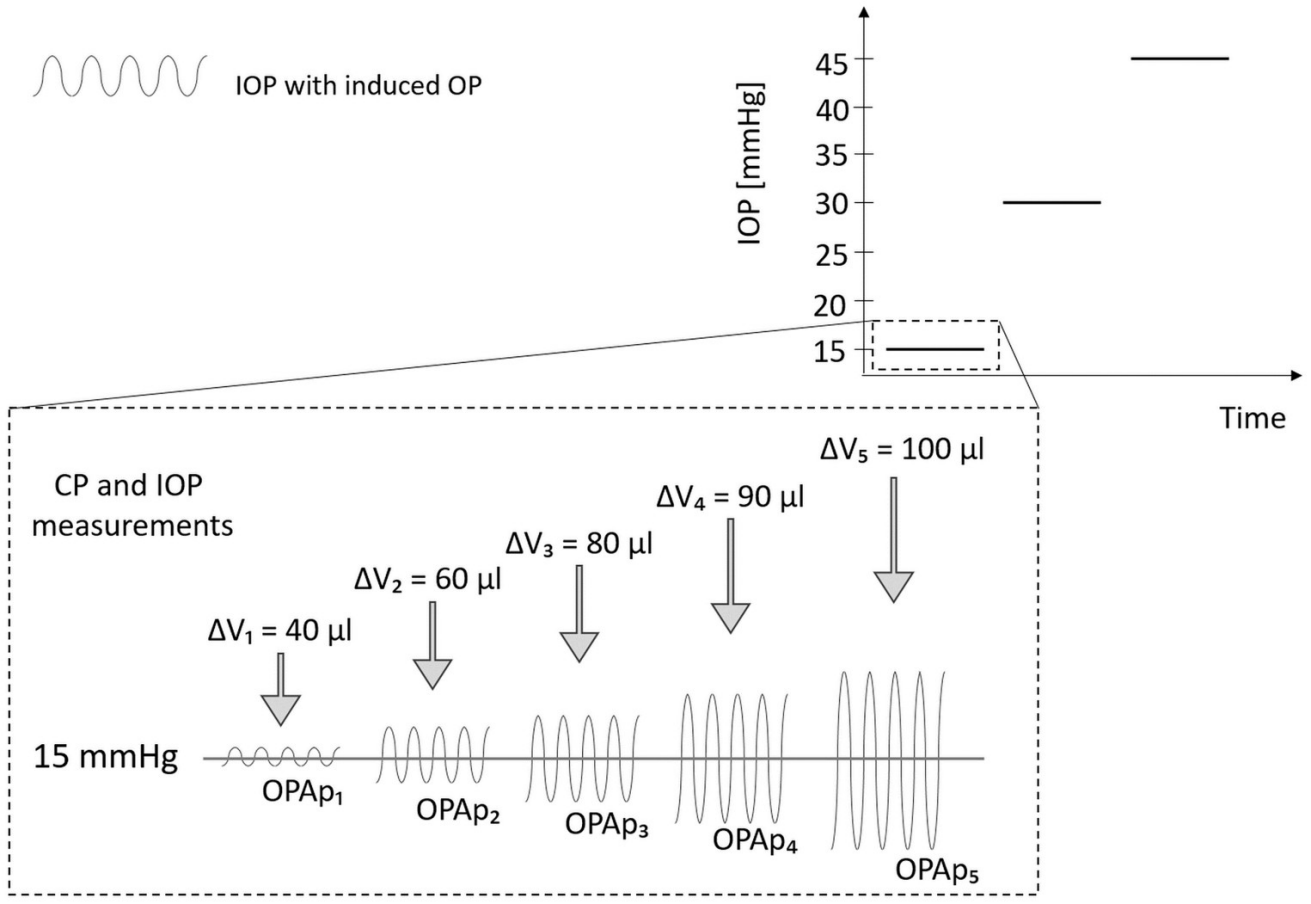
A scheme of the CP and IOP signal measurement procedure during ocular pulse (OP) simulations with controlled infusion/withdrawal PBS volume (ΔV) in the three-step inflation experiment.

Two experiments were considered. In the first one, a selected eye globe was subjected to three levels of IOP (15, 30, and 45 mmHg), where the OP was generated as a sine wave with a frequency of 1.2 Hz and an amplitude, OPAp, corresponding to controlled infusion/withdrawal PBS volume forced by the pump. The test was performed for the five different volumes (ΔV): 40, 60, 80, 90 and 100 μl (Fig 2) set at each IOP value. OPAp stands for the physical ocular pulse amplitude value [16] and is the difference between peak and trough in the pressure signal.

During all the enabled OP simulations, the CP signal was acquired in a non-contact manner using ultrasonic distance sensor (UltraLab, Wrocław, Poland) [46]. Synchronically with the CP signal, the IOP signal was registered with the pressure transmitter. The three 10-second length continuous data acquisitions were performed at each setting.

The second experiment involved a number of eye globes which were subjected to IOP increased from 15 mmHg to 45 mmHg in steps of 5 mmHg. The infusion/withdrawal volume was adjusted to obtain pressure amplitude value at about 4 mmHg (mean ± SD, 4.25 ± 0.25 mmHg) for the IOP equal to 15 mmHg, which gives from about 70 μl to 90 μl of PBS. The determined volume was maintained at each IOP value for the given eye that, in turn, should lead to a natural increase of OPAp as a result of the increase of IOP according to the results obtained from previous in-vivo animal and human studies [7,55]. This effect is due to the decreasing ability of the ocular tissue to deform under higher internal pressure.

**Fig 3:**
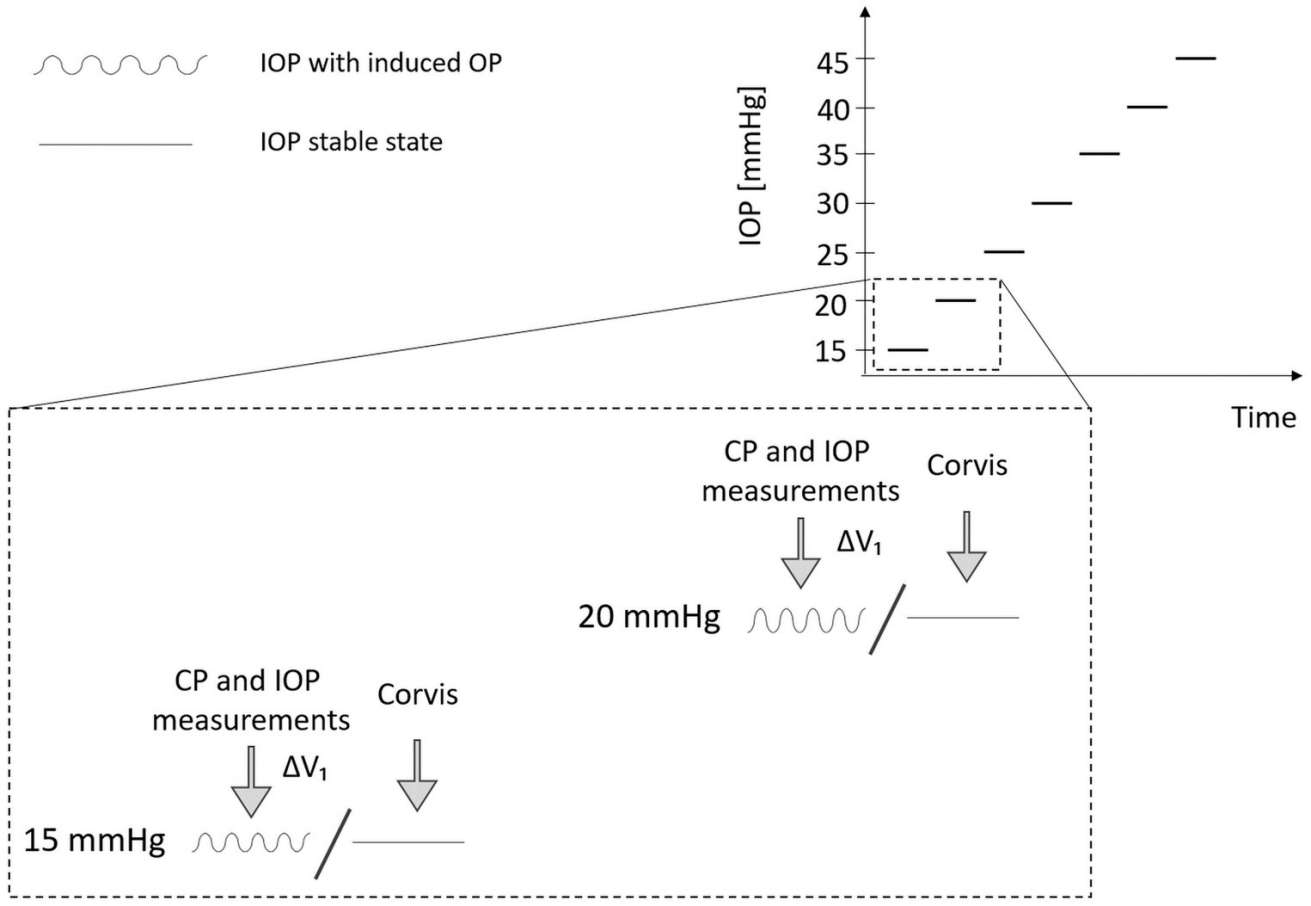
A scheme of the CP and IOP signal measurement procedure during ocular pulse (OP) simulations with constant infusion/withdrawal PBS volume (ΔV) together with corneal biomechanical measurements in the seven-step inflation experiment.

At each IOP, when the OP simulation was enabled, the CP and IOP signals were registered as in the first experiment. Also, in this experiment, static cases of IOP (without OP) were investigated (see Fig 3). When induction of OP was disabled, three corneal biomechanical measurements were performed using a dynamic Scheimpflug analyzer, Corvis ST (Oculus, Optikgeräte GmbH, Wetzlar, Germany). The device provided 10 corneal deformation parameters that were included in the analysis: the time from the start until the first/second applanation (A1T/A2T), the length of the flattened cornea during the first/second applanation (A1L/A2L), the corneal velocity at the two applanation events (A1V, A2V), and the four parameters describing the moment of the maximum deformation of the cornea: the amplitude (DA), the distance between corneal bending points (PD), the time from the start until the highest concavity of cornea (HCT), and the radius of the curvature at the corneal apex (R).

### Signal analysis

The CP and IOP signals were acquired at a sampling frequency of 400 Hz. CP was determined with the accuracy below 1 μm whereas IOP with a precision of 0.1 mmHg. Both signals were numerically processed using custom written program in MATLAB (MathWorks, Inc. Natick, MA, USA). In the case of the CP signal linear trend was removed. Then, as in the case of OPAp, the corneal pulse amplitude (CPA) was determined as the difference between the median value of successive peaks and the median value of the successive troughs in the signal.

In the second experiment, the considered parameters from the three measurements assigned to the specific IOP value were averaged and then normalized to their mean value calculated for the baseline IOP of 15 mmHg for each eye.

All experimental data are included in the S1 File of Supporting information.

### Biomechanics

In order to estimate the stiffness of the corneal tissue in the particular experimental setup considered here, a new index named *ocular pulse based corneal rigidity index* (OPCRI) is introduced for the OP cycles. The methodology is analogous to the conventional calculation of the Young modulus *E*, however, it solves the problem of the inability to assess the exact value of *E* without extracting corneal tissue from the eye globe. The CPA and OPAp were taken as surrogate data for the stress-strain relationship, where OPAp, as an internal pressure change, substitutes the stress value, while CPA, as a corneal surface expansion, is the indirect measure of the resulting strain. Consequently, the higher the OPCRI value is obtained the more pressure is needed to yield a change in the ocular volume. Here, OPCRI was used to determine the corneal tissue stiffness at different IOP.

### Statistical analysis

Statistical analyses were conducted using Statistica (StatSoft, Inc., USA). Firstly, in order to find if the experimental data meet the assumptions for parametric testing the Shapiro–Wilk W test of normality and Levene’s test for equality of variances were applied. Because not all of the parameters fulfilled the prerequisites of parametric test procedures the Friedman ANOVA test was employed to ascertain if the IOP statistically significantly differentiate the CP signal parameters and those describing the corneal biomechanics. In addition, depending on the Fisher’s exact test results, either linear or exponential modelling was used for those relationships. Furthermore, partial correlation was performed to investigate the dependencies between CPA or OPCRI and corneal deformation parameters, with IOP set as a control variable. The significance level α was set to 0.05 for all tests used in this study.

## Results

Six porcine eye globes passed the inclusion criteria. The ocular biometric parameters with its mean and median values are gathered in the Table 1.

**Table 1:** Summary of data.

Parameter	Mean ± SD	Median	Range
CCT [μm]	1067 ± 57	1061	[982, 1165]
R [mm]	8.10 ± 0.24	8.13	[7.74, 8.53]
LT [mm]	7.79 ± 0.37	7.99	[7.05, 8.05]
ACD [mm]	3.07 ± 0.27	3.14	[2.67, 3.48]
AQD [mm]	2.01 ± 0.25	2.12	[1.66, 2.31]
AL [mm]	21.02 ± 0.58	20.80	[20.40, 22.22]

Central corneal thickness (CCT), corneal radius (R), lens thickness (LT), anterior chamber depth (ACD), aqueous humor depth (AQD), axial length (AL).

**Fig 4:**
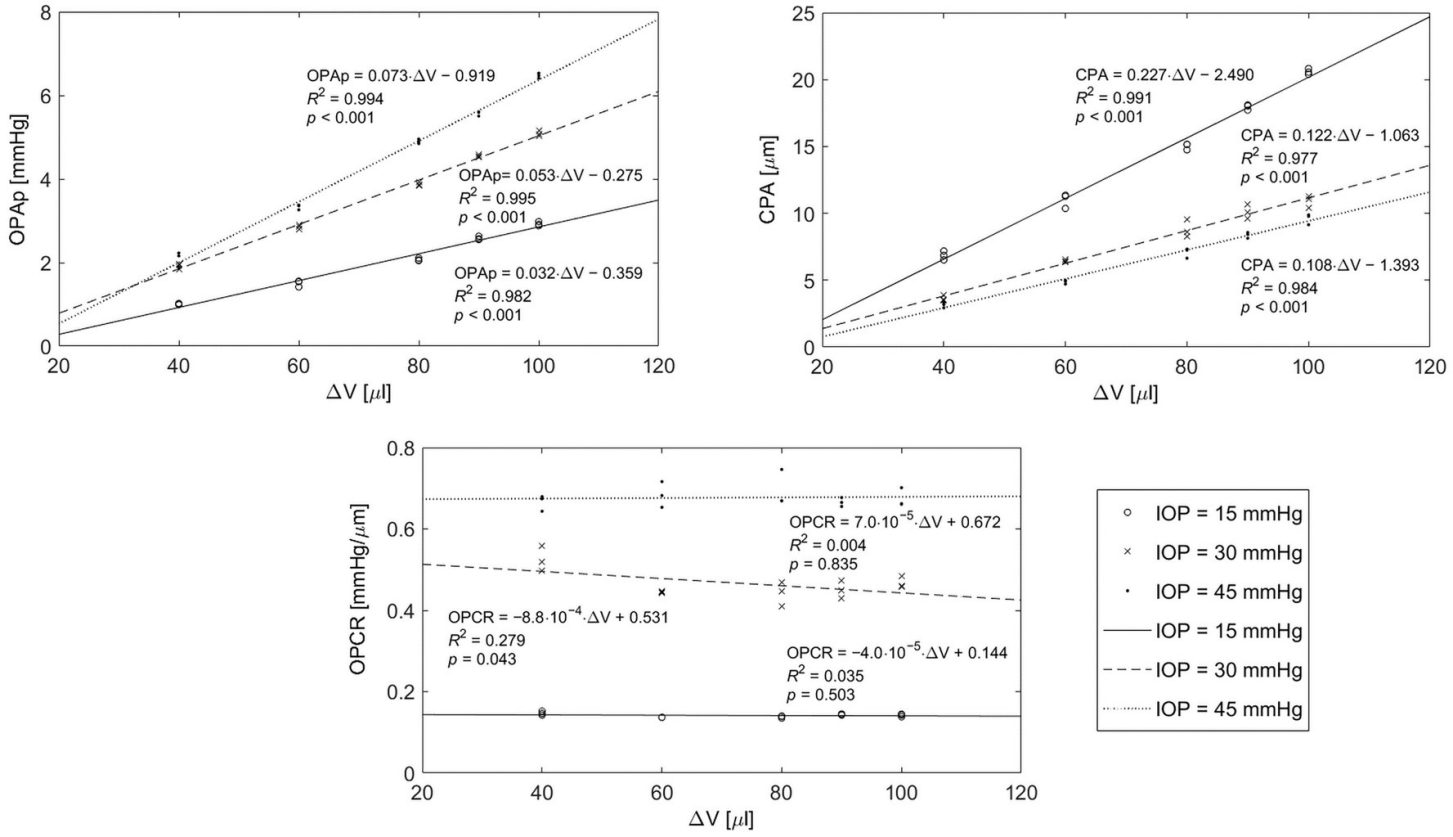
Regression analysis of physical ocular pulse amplitude (OPAp), corneal pulse amplitude (CPA), and ocular pulse based corneal rigidity index (OPCRI), with infusion/withdrawal volume ΔV, for three different IOP values.

The first experiment showed that the elevation of IOP results in an increase of physical ocular pulse amplitude (OPAp) for the same infusion/withdrawal volume. Fig 4 shows the linear models of OPAp versus ΔV for each IOP value. The regression coefficients obtained for IOP = 15, 30 and 45 mmHg are 0.03, 0.05, and 0.07 mmHg/μl, respectively. Similar effect was observed for corneal pulse amplitude (CPA) (Fig 4). However, in this case corneal tissue response to changes in ΔV is weaker after raising IOP (the subsequent regression coefficients are: 0.23, 0.12 and 0.11 μm/μl).

An increase in OPCRI can be observed with the increase in IOP. However, OPCRI remains stable at the particular IOP (regression coefficients: −0.00004, −0.00088, and 0.00007 mmHg/(μm·μl) for IOP = 15, 30, and 45 mmHg, respectively) (see Fig 4).

**Fig 5:**
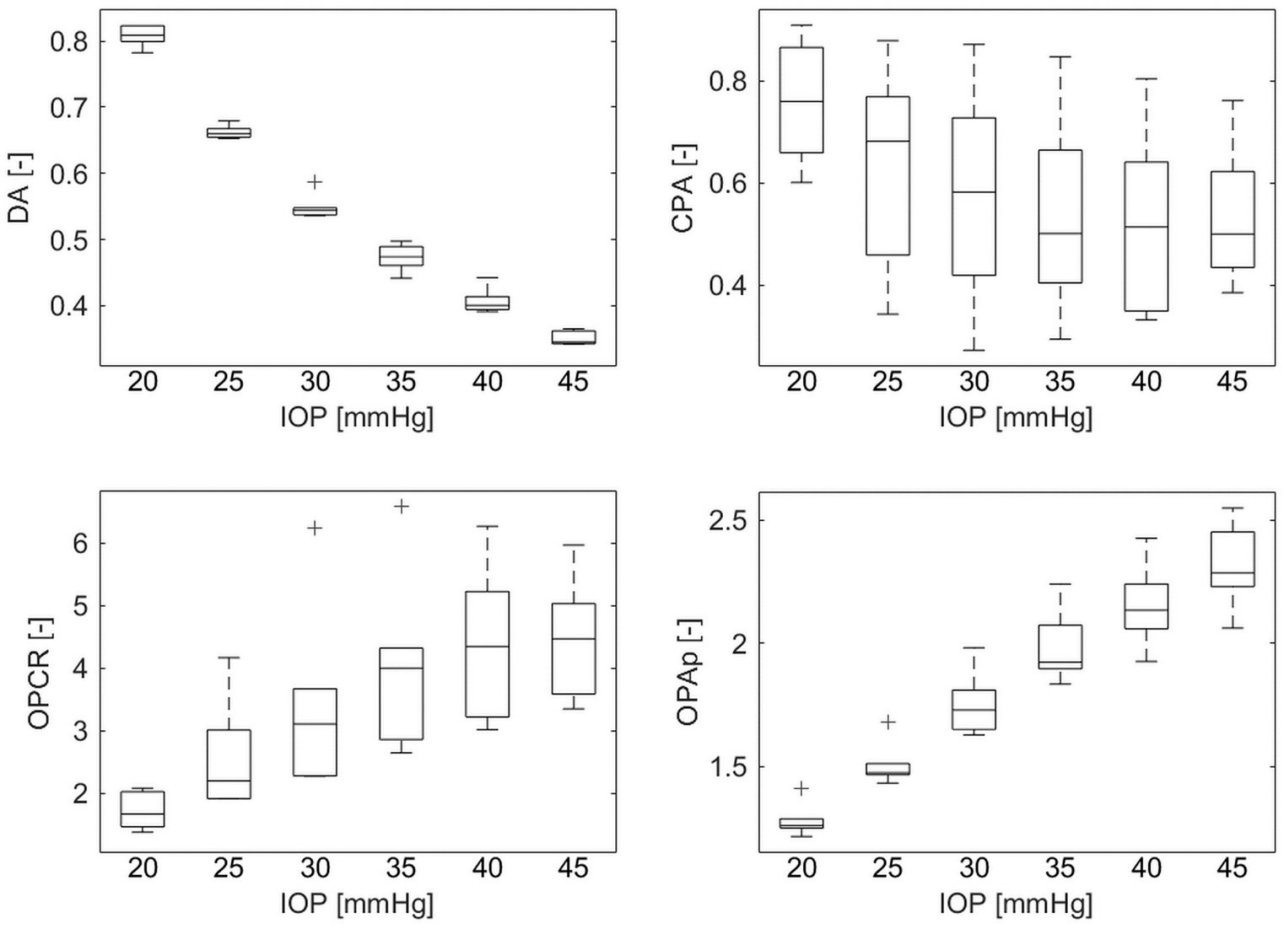
Boxplots of deformation amplitude (DA), corneal pulse amplitude (CPA), ocular pulse based corneal rigidity index (OPCRI), and physical ocular pulse amplitude (OPAp) normalized to their baseline values obtained at 15 mmHg, for different IOP values.

In the second experiment, significant differences were present among the IOP in the changes of almost all the corneal deformation parameters (Friedman ANOVA, *p* < 0.05). The only exceptions were the A1L (Friedman ANOVA, *p* = 0.416), A2L (Friedman ANOVA, *p* = 0.087), and HCT (Friedman ANOVA, *p* = 0.208). IOP significantly differentiate also OP parameters (CPA and OPAp) and OPCRI (Friedman ANOVA, *p* = 0.013, *p* < 0.001, *p* < 0.001, respectively) (Fig 5). Table 2 summarizes these results.

**Table 2:** Results of the Friedman ANOVA test presenting IOP impact on the changes in the corneal deformation parameters and the ocular pulse signal parameters. In addition, depending on the Fisher’s exact test outcome, either linear or exponential model was applied.

Normalized parameter	Friedman ANOVA		Linear model		Exponential model		Goodness of fit			Fisher test (linear vs. exponential)
			*f*(*IOP*) = *a* ∙ *IOP* + *b*		*f*(*IOP*) = *c* ∙ *exp*(*d* ∙ *IOP*)					
	Chi Sqr.	*p*-value	*a*	*b*	*c*	*d*	SSE	R^2^	RMSE	*p*-value
CPA [-]	14.38	**0.013**	−0.0085	0.87	-	-	1.02	0.16	0.18	0.834
OPAp [-]	30.00	**<0.001**	-	-	0.87	0.022	0.69	0.87	0.15	**0.013**
OPCRI [-]	26.00	**<0.001**	0.11	−0.26	-	-	41	0.46	1.1	0.296
A1L [-]	5.00	0.416	0.0019	1.01	-	-	0.35	0.028	0.10	0.996
A1V [-]	29.15	**<0.001**	-	-	1.52	−0.034	0.21	0.80	0.078	**0.016**
A2L [-]	9.62	0.087	0.010	1.13	-	-	4.6	0.060	0.37	0.978
A2V [-]	29.24	**<0.001**	-	-	2.73	−0.063	0.081	0.95	0.049	**< 0.001**
PD [-]	28.76	**<0.001**	−0.020	1.42	-	-	0.69	0.60	0.15	0.581
R [-]	28.38	**<0.001**	0.022	0.65	-	-	0.43	0.75	0.12	0.555
DA [-]	30.00	**<0.001**	-	-	1.57	−0.034	0.012	0.99	0.019	**< 0.001**
A1T [-]	30.00	**<0.001**	-	-	0.74	0.016	0.059	0.95	0.042	**< 0.001**
HCT [-]	7.17	0.208	−0.00087	0.99	-	-	0.017	0.11	0.023	0.992
A2T [-]	30.00	**<0.001**	−0.0035	1.05	-	-	0.0035	0.90	0.01	0.741

The results of partial correlation are shown in Table 3. Both CPA and OPCRI correlate with A1L, whereas only CPA correlates with A2T.

**Table 3:** Results of partial correlation between normalized corneal pulse amplitude (CPA) or ocular pulse based corneal rigidity index (OPCRI) and normalized corneal deformation parameters, with IOP used as a control variable.

Normalized parameter	Control variables		A1L	A1V	A2L	A2V	PD	R	DA	A1T	HCT	A2T
**CPA**	*IOP*	*r*	**−0.506**	0.024	0.007	−0.117	0.310	−0.286	0.213	−0.199	−0.172	**0.414**
		*p*	**0.002**	0.892	0.968	0.503	0.070	0.096	0.220	0.252	0.324	**0.014**
**OPCRI**	*IOP*	*r*	**0.482**	−0.009	−0.091	0.155	−0.264	0.078	−0.116	0.047	0.039	−0.331
		*p*	**0.003**	0.959	0.605	0.375	0.125	0.657	0.506	0.788	0.825	0.052

## Discussion

The pressure-volume relationship in the anterior chamber is of particular interest for finding the effective method for *in-vivo* measuring ocular stiffness and for accurate IOP reading, which is crucial in diagnostics of ophthalmic diseases [18,41]. The pressure-volume dynamics is strictly related to IOP fluctuations and biomechanics of ocular tissues [7]. The pulsatile component of IOP comes from the pulsatile ocular blood flow induced by the arterial blood pulse [34]. The difference between the systolic and diastolic IOP values in the IOP characteristic is named the ocular pulse amplitude (OPA), whose average values range from 2 mmHg to 4 mmHg in healthy subjects [56–58]. In clinical practice, the dynamic contour tonometer (DCT; Pascal tonometer) is the only currently commercially available instrument to *in-vivo* measure the IOP wave in a contact manner after the cornea anesthesia [59]. This measurement procedure prevents the natural expansion of the cornea and influences corneal biomechanical properties which are crucial to accurately estimate the IOP. This study contributes to this knowledge by providing information on the relationship between corneal pulsation and the IOP and its fluctuations during the *ex-vivo* IOP elevation experiment with porcine eyes with artificially induced OP cycles using the ultrasonic technique which enables registering natural corneal pulsation without any external stimuli and tissue contact. The use of porcine eyes was dictated by the fact that they share many anatomical and physiological similarities to those of the human eye [60–62]. In many *ex-vivo* studies related to biomechanical properties of eye tissues, the porcine cornea can be used as a substitute model for human cornea study, according to the stress-strain pattern [42,63,64].

For the first *ex-vivo* inflation experiment, the results show that both physical ocular pulse amplitude (OPAp) and corneal pulse amplitude (CPA) positively correlate with infusion/withdrawal volume. However, the IOP elevation magnifies the relationship between changes in the internal pressure and those in the ocular volume (OPA versus ΔV) and, simultaneously, weakens the corneal tissue response to the changes in ocular volume (CPA versus ΔV). An increase of OPCRI—an index that was found to be uncorrelated with ΔV—at elevated IOP confirmed that biomechanical properties of the cornea change with the applied stress leading to rigidity increase. Hence, the corneal stiffness growth, concurrent with the IOP raise, prevents tissue ability to deform (represented by CPA) but also causes an increase in the amplitude of IOP changes (represented by OPAp). These findings are in agreement with a study by Clayson et al. [20] on porcine eyes, which showed that the induced corneal stiffening, obtained by crosslinking treatment, and the IOP rise significantly impact the magnitude of IOP spikes. In human studies, the positive correlation between IOP and OPA has been also reported in healthy subjects [58] as well as in patients with ophthalmic diseases [7,19] showing increased mechanical resistance of the ocular wall at high IOP.

In the second *ex-vivo* experiment, the IOP impact on corneal deformation parameters and OP signal parameters was assessed. The results of corneal deformation obtained using Corvis ST are consistent with those of Bao and colleagues [16] showing that the majority of biomechanical metrics are correlated with IOP and their relationship can be well described using either linear or exponential model (see Table 2).

In the current study, a new approach, which additionally takes OP signal parameters into account, has shown that OP signal parameters are correlated with IOP. When comparing CPA and deformation amplitude changes with IOP rise, one can clearly see that increasing the internal load reduces corneal ability to expand naturally during OP cycles and, at the same time, to deform in response to the air puff. This conclusion is consistent with OPCRI behavior under incremental rise of IOP, highlighting the increase in corneal rigidity with the load, as it has also been shown in the first experiment.

The OPAp has been found to increase with IOP rise, even though it was triggered with the constant ΔV at all IOP values. This observation confirms the results obtained in the first experiment of this study and also corresponds to the previous *in-vivo* animal [55] and human [7] studies demonstrating the increased OPA with artificial increase in IOP. Dastiridou and colleagues [7] suggested that a higher OPA may appear in eyes with increased ocular rigidity.

Ocular rigidity is usually obtained for the whole pressure-volume characteristic of the eye, which is approximated as a linear [65] or exponential model [7]. In this paper, the rigidity index, OPCRI, was introduced as a measure of the corneal response to the induced internal pressure pulsation imitating OP. This means that OPCRI calculation is focused on the small recurring changes of the load rather than a single inflation event. It is worth noting that OPCRI is based on corneal pulsation measured without any external stimulation, in contrast to Corvis ST, where complete applanation cycle of the cornea is required to acquire this information. OPCRI enables simple and indirect estimation of the tissue rigidity at different IOP. The study shows that the relationship between IOP and corneal rigidity in the form of OPCRI, as well as between IOP and CPA, can be modelled in a linear fashion. It is worth emphasizing that OPCRI is not the only alternative (with respect to Corvis ST) approach to estimating corneal stiffness. Another measure, termed ocular pulse stiffness index (OPSI), based on induced IOP pulsation was proposed by Pavlatos and colleagues [31]. The OPSI was introduced to verify effectiveness of the ocular pulse ultrasound elastography aiming at evaluating corneal biomechanics on the basis of naturally occurring OP and without the necessity of exerting external force needed to induce tissue deformation. However, the main difference between OPSI and OPCRI lies in the applied ultrasound techniques, which, in the case of OPCRI, allows registering corneal surface pulsation in an air-coupled manner without disturbing natural corneal dynamics. Worth noting is that both techniques are complementary as one provides high spatial resolution whereas the other provides high temporal resolution.

In this study, the relationships between corneal deformation parameters and OP signal parameters have been examined taking into account the influence of IOP on these parameters (please refer to Table 3). The results indicate that A1L correlates with both CPA and OPCRI, underlining the role of corneal biomechanical properties in changes of natural corneal pulsation. Recent studies have revealed that the applanation length is mostly affected by CCT and age of the subjects rather than by a change of IOP [66]. Hence, it was suggested that A1L could be used to determine the stiffness of the cornea. In the light of the above, the current study demonstrates that CPA values could be utilized for estimating, in an indirect way, corneal biomechanical changes linked to the higher corneal stiffness.

Limitations of this study include potential postmortem reactions of the *ex-vivo* specimens, e.g., corneal swelling, on the resulting analysis. Special effort was taken in order to minimize those effects during the experimental procedure. Nevertheless, the amount of any bias resulted from corneal swelling is likely to be the same for all considered cornea. Eusol-C and mineral oil were applied to maintain proper hydration of the tissue. The study relies on *ex-vivo* porcine eye globes substituting human eyes, which are difficult to obtain from human donors. Even so, engaging porcine model proved to be quite problematic. Some elements of the procedures used at abattoir may result in optical or mechanical defects of the eye globes, e.g., corneal opacity or impaired corneal epithelium. Therefore, only very limited number of porcine eye globes satisfied the inclusion criteria. In that sense, our study has a pilot character. In the first experiment, increasing infusion/withdrawal PBS volume at the particular IOP value, with a frequency maintained constant, results in both raise of the OPAp and acceleration of the pressure change during OP cycles. This small variations of the velocity of the load were neglected in this study, however, we are conscious that this aspect is important in the context of viscoelastic properties of the cornea [10,67]. Constant frequency of the OP simulation was set to reflect typical heart rhythm in a healthy human.

## Conclusions

Summarizing, this study revealed that the corneal biomechanical changes related to increase in IOP can be detected by observing natural corneal pulsations with a non-contact ultrasonic technique. Specifically, it was shown that the increase in IOP fluctuations and corneal stiffness co-occurring with the IOP growth could be estimated indirectly based on the corneal pulse amplitude. The newly proposed rigidity index, calculated on the basis of the CP signal, could be an approximate estimator of the corneal stiffness corresponding to both IOP variations and changes in biomechanical properties of the cornea. More importantly, unlike in elastography, this knowledge can be acquired without disturbing natural corneal dynamic.

## Supporting information

S1 File Data set. (XLSX)
